# Denatured G-Protein Coupled Receptors as Immunogens to Generate Highly Specific Antibodies

**DOI:** 10.1371/journal.pone.0046348

**Published:** 2012-09-27

**Authors:** Franck Talmont, Lionel Moulédous, Jérôme Boué, Catherine Mollereau, Gilles Dietrich

**Affiliations:** 1 CNRS, IPBS (Institut de Pharmacologie et de Biologie Structurale), Toulouse, France; 2 Université Paul Sabatier Toulouse III, Toulouse, France; 3 INSERM, U1043, Centre de Physiopathologie de Toulouse-Purpan, Toulouse, France; 4 CNRS, U5282, Toulouse, France; Institute of Molecular and Cell Biology, Singapore

## Abstract

G-protein coupled receptors (GPCRs) play a major role in a number of physiological and pathological processes. Thus, GPCRs have become the most frequent targets for development of new therapeutic drugs. In this context, the availability of highly specific antibodies may be decisive to obtain reliable findings on localization, function and medical relevance of GPCRs. However, the rapid and easy generation of highly selective anti-GPCR antibodies is still a challenge. Herein, we report that highly specific antibodies suitable for detection of GPCRs in native and unfolded forms can be elicited by immunizing animals against purified full length denatured recombinant GPCRs. Contrasting with the currently admitted postulate, our study shows that an active and well-folded GPCR is not required for the production of specific anti-GPCR antibodies. This new immunizing strategy validated with three different human GPCR (μ-opioid, κ-opioid, neuropeptide FF2 receptors) might be generalized to other members of the GPCR family.

## Introduction

G-protein coupled receptors are involved in many key biological processes such as smell, taste and vision. These membrane proteins mediate responses to hormones, neurotransmitters, metabolites, ions, fatty acids, pathogens and physical stimuli. Based on the human genome sequencing, 60% of the 800 GPCRs that have been identified belong to the so-called olfactory or sensory receptors. The remaining 40% are classified in five main families under the GRAFS system (Glutamate, Rhodopsin, Adhesion, Frizzled/taste2 and Secretin) [1], [2]. In line with their pivotal role in a number of physiological processes, GPCRs have been found dysregulated in several human pathologies including cardiovascular and gastrointestinal diseases, nervous and immune disorders and cancers. As a matter of fact, nearly half of the drugs marketed by pharmaceutical industries targets GPCRs.

In this context, highly specific anti-GPCR antibodies may be particularly helpful to better define anatomical localization as well as biochemical and biological properties of the receptors targeted for therapy [3]. Antibodies may be used to reveal GPCR expression on living cells (as assessed by cytofluorometry or confocal microscopy) or on membrane extracts (Western blotting) as well as *in situ* on fixed tissue sections (immunochemistry). Specific antibodies may be helpful to purify receptors [4], characterize receptor dimers [5], identify receptor-associated protein partners [6] (immunoprecipitation), stabilize GPCR for crystallography [7], study ligand-binding kinetics [8] and conformation states [9]. In the absence of specific ligands, anti-GPCR antibodies are a valuable alternative for studying orphan receptors. Moreover, development of antibodies against GPCRs such as adhesion receptors, for which conventional small molecule drug discovery methods are often unsuccessful, offers a promising alternative for pharmaceutical industries. Approximately 80 GPCRs, notably those involved in cancer, inflammatory or metabolic disorders have been recently identified as suitable targets for antibody-based therapy [10]. Anti-GPCR antibodies, that do not cross the blood-brain barrier because of their high molecular weight, could also be instrumental in only targeting GPCRs expressed in periphery. Thus, agonistic antibodies with no central nervous system-mediated side effects might be used to relieve from inflammatory pain by stimulating opioid receptors expressed on sensory neurons [11], [12], [13], [14].

Specific antibodies against a variety of antigens including GPCRs can be developed using phage display technology [15], but the common method to produce antibody probes consists in immunizing animals against target proteins. As a matter of fact, most of the available anti-GPCR antibodies are polyclonal serum IgG generated by immunizing animals with synthetic peptides corresponding to amino-acid sequences located within the amino (extracellular)–terminal or carboxy (intracellular)-terminal domains or within extra- or intra-cellular loops of the receptors. However, as recently reported for a number of GPCRs including opioid receptors [16], commercial available polyclonal antibodies often display non-specific reactivities and/or cross-reactivities with other plasma membrane proteins thus making it difficult to clearly distinguish a specific antibody-receptor binding. In most of the cases, the staining patterns of anti-GPCR peptide antibodies are similar in wild-type and GPCR-deficient mice as assessed by immunohistochemistry or western-blotting [16], [17], [18], [19], [20], [21], [22]. A recent study, comparing the specificity of a number of commercial anti-opioid receptor antibodies, has shown that all the antibodies revealed numerous non-specific bands including a band at the expected molecular weight in both wild-type CHO cells (negative control) and GPCR-expressing CHO cells as assessed by western-blotting [23]. Given the lack of specificity of anti-GPCR peptide antibodies, it is now generally accepted that the production of relevant anti-GPCR antibodies able to recognize native proteins requires immunizing animals with receptors in native conformation, thus maintaining their ligand-binding activity [7], [10], [24], [25]. The hydrophobic nature of GPCRs and their low natural expression make, however, the purification of high amounts of native-like functional receptors a formidable challenge [26], [27]. Considering the technical skills that are needed to obtain GPCRs in native form, the peptide strategy is still used by many laboratories despite its low success level.

Here, we describe a novel strategy to easily produce highly specific anti-GPCR antibodies by using purified denatured full length recombinant GPCRs, as immunogens. We show an unanticipated finding that correctly folded native-like receptors are not required to produce highly specific antibodies to GPCRs. This method successfully applied to the human neuropeptide FF receptor type 2 (hNPFFR_2_), the human κ opioid receptor (hKOR) and the human μ opioid receptor (hMOR) might be extended to a wide range of other GPCRs and applicable in most laboratories.

## Results

### Immunogen preparation and immunization

**Figure 1:**
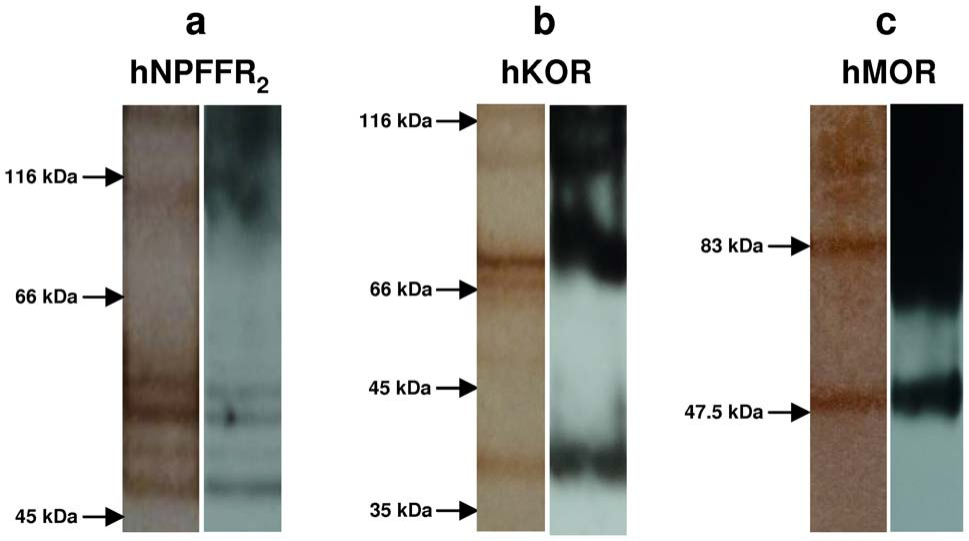
Purity of GPCR-enriched samples used as immunogens. Recombinant hNPFFR_2_ (a), hKOR (b) and hMOR (c) with six histidine residues and a c-myc tag fused to their C-terminus were over-expressed in *Pichia Pastoris*. GPCR-enriched crude fractions obtained by differential centrifugation of GPCR-expressing yeast cell lysates were solubilized and then chromatographed on a nickel affinity column. Ni-bound GPCRs were eluted with imidazole-containing buffer. GPCR-enriched samples run in SDS-polyacrylamide gels were stained by silver nitrate (left panels) or probed with anti-c-myc monoclonal antibody after transfer onto PVDF membrane (right panels).

Recombinant human G-protein coupled receptors with six histidine residues and a c-myc tag fused to their C-terminus were produced in the methylotrophic yeast *Pichia Pastoris*. Receptors were solubilized in 0.1% sodium dodecyl sulphate (SDS) and 8 M urea and subsequently chromatographed upon nickel affinity column. Receptors that bound to nickel-agarose phase because of their C-terminal histidine residues were eluted using an imidazole gradient buffer [28]. Purity of each GPCR-enriched preparation was assessed by comparing silver nitrate staining of SDS-PAGE gels with an anti-c-myc immunoblotting (**Fig. 1**). All proteins stained by silver nitrate were also revealed with anti-c-myc antibodies indicating that the three GPCR preparations used to immunize mice were virtually pure. Silver nitrate dyeing as well as anti-c-myc staining, revealed bands with apparent molecular weights of 47 kDa, 40 kDa and 47.5 kDa corresponding to the calculated size of hNPFFR_2_ (**Fig. 1a**), hKOR (**Fig. 1b**) and hMOR (**Fig. 1c**) respectively (**Table 1**). Bands with higher molecular weights were also stained with both silver nitrate and anti-c-myc antibodies. Thus, the preparations of recombinant GPCRs purified from *Pichia Pastoris* contain receptors in unglycosylated monomeric forms but also in polymeric and/or glycosylated forms as described elsewhere [29], [30], [31]. Receptor preparations depleted in their imidazole content (to minimize toxicity) were then either maintained in solubilized form in 0.1% SDS or dialyzed against water and lyophilized. The two kinds of receptors preparations were used to immunize animals. BALB/c mice were injected subcutaneously with 100 µg of purified receptors emulsified in complete Freund's adjuvant followed by two injections two weeks apart with the same amounts of proteins in incomplete Freund's adjuvant. For each GPCR preparation (*i.e.* in water and in SDS), two sets of immunization were performed on three or four animals.

**Table 1:** Characteristics of human G-protein coupled receptors used to generate immune serum IgG antibodies.

Receptor	Gene	Accession number	Size (AA)	Theoretical Molecular weight (kDa)
hMOR	*OPRM1*	NP 000905	400	44.78
hKOR	*OPRK1*	NP 000903	380	42.65
hNPFFR_2_	*NPFF2*	NP 444264.1	420	48.69

AA: amino acids.

kDa: kilodalton.

### Detection of receptors expressed in recombinant cells

**Figure 2:**
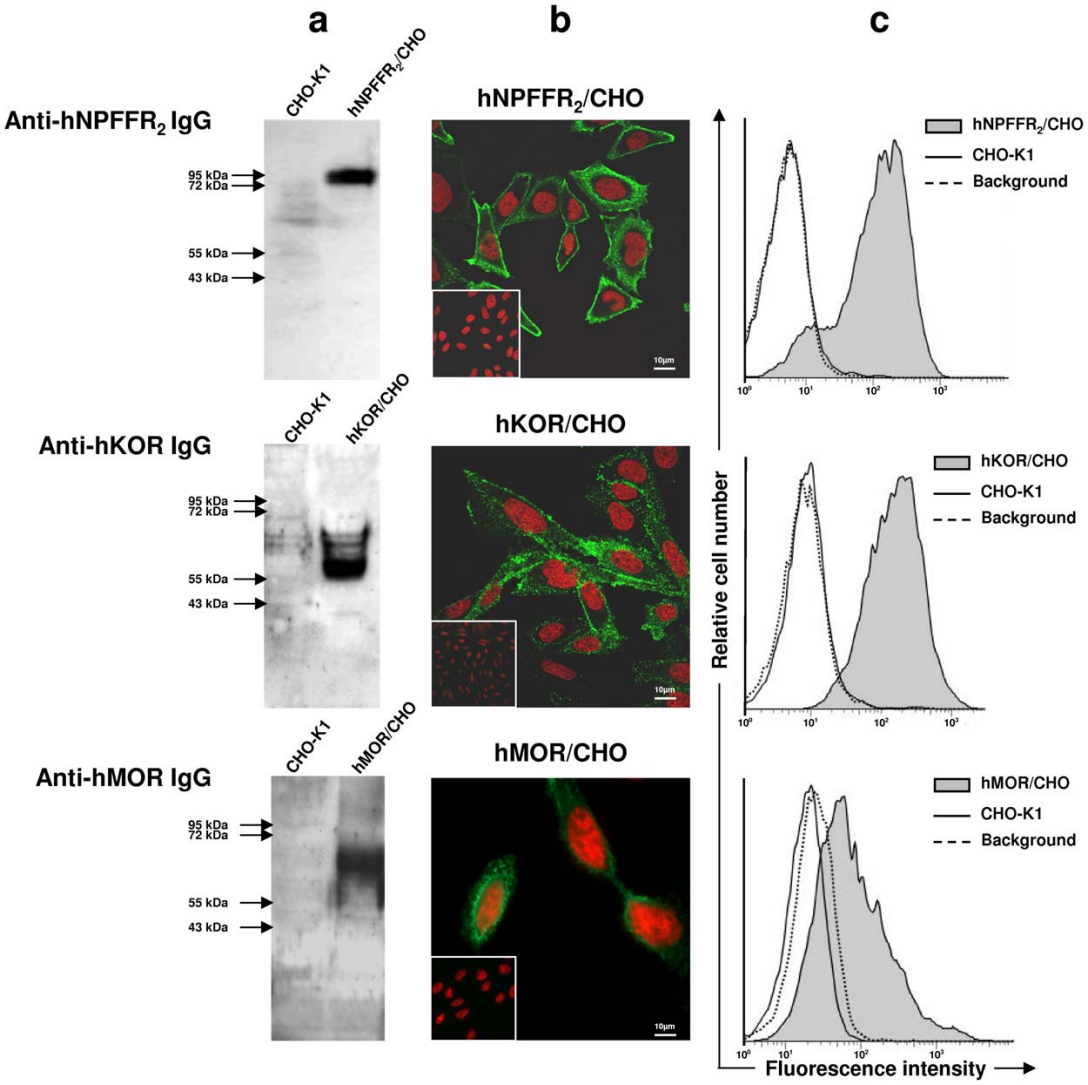
Binding of immune serum IgG to recombinant wild-type GPCRs expressed in CHO cells. The ability of immune serum IgG from mice immunized against hNPFFR_2_ (upper panels), hKOR (middle panels) and hMOR (lower panels) to specifically bind to their corresponding receptors (*i.e.* used for immunization) was assessed by western-blotting (a), confocal immunofluorescence microscopy (b), and cytofluorometry (c). In (a), membrane proteins extracted from untransfected CHO-K1 cells (left lanes) or from CHO-K1 cells expressing either hNPFFR_2_ (hNPFFR_2_/CHO), hKOR (hKOR/CHO) or hMOR (hMOR/CHO) (right lanes) were run on SDS-polyacrylamide gel and transferred onto PVDF membrane. Protein extracts were probed with corresponding mouse immune sera. Bound IgG were revealed using horseradish peroxidase-labeled rabbit anti-mouse IgG antibodies. In (b), CHO cells expressing wild-type GPCR including hNPFFR_2_/CHO, hKOR/CHO, hMOR/CHO or CHO-K1 cells (insert) were incubated with immune sera collected from mice immunized against the corresponding receptor. Bound IgG were then revealed with Alexa 488-labeled goat anti-mouse IgG antibodies (green staining). Cell nuclei were stained in red with propidium iodide. Fluorescence images were acquired by confocal microscopy. In (c), the binding of immune serum IgG to CHO cells expressing their corresponding receptors was examined by cytofluorometry: hNPFFR_2_/CHO cells (upper panel), hKOR/CHO cells (middle panel) and hMOR/CHO cells (lower panels). GPCR-expressing CHO cells (grey histogram) and CHO-K1 cells (open histogram) were incubated with immune sera for 30 min at 4°C. Bound IgG were then revealed with biotin-conjugated goat anti-mouse antibodies followed by an additional incubation with allophycocyanin-labeled streptavidin. Backgrounds (dotted line) correspond to GPCR-expressing CHO cells or wild-type CHO-K1 cells stained with normal serum IgG from non-immunized mouse. The figure shows one representative experiment out of 3 performed.

The antibody specificity of serum IgG collected from immunized mice was first examined by western-blotting on the wild-type recombinant receptors without c-myc tag fused to the C terminus. The ability of polyclonal antibodies (serum dilution ranging from 1/500 to 1/4000) to specifically recognize receptors was assessed by comparing their immunodetection in extracts from membrane of CHO-K1 cells expressing the relevant GPCR (1–10 pmol/mg membrane proteins) and from wild-type CHO-K1 cells. For each receptor, a unique band was revealed by immune serum IgG antibodies as assessed by western-blotting (**Fig. 2a**). Similar results were obtained with all individual immune sera from mice immunized with GPCRs both in water and 0.1% SDS. No IgG binding to control CHO-K1 cell membranes was observed.

**Figure 3:**
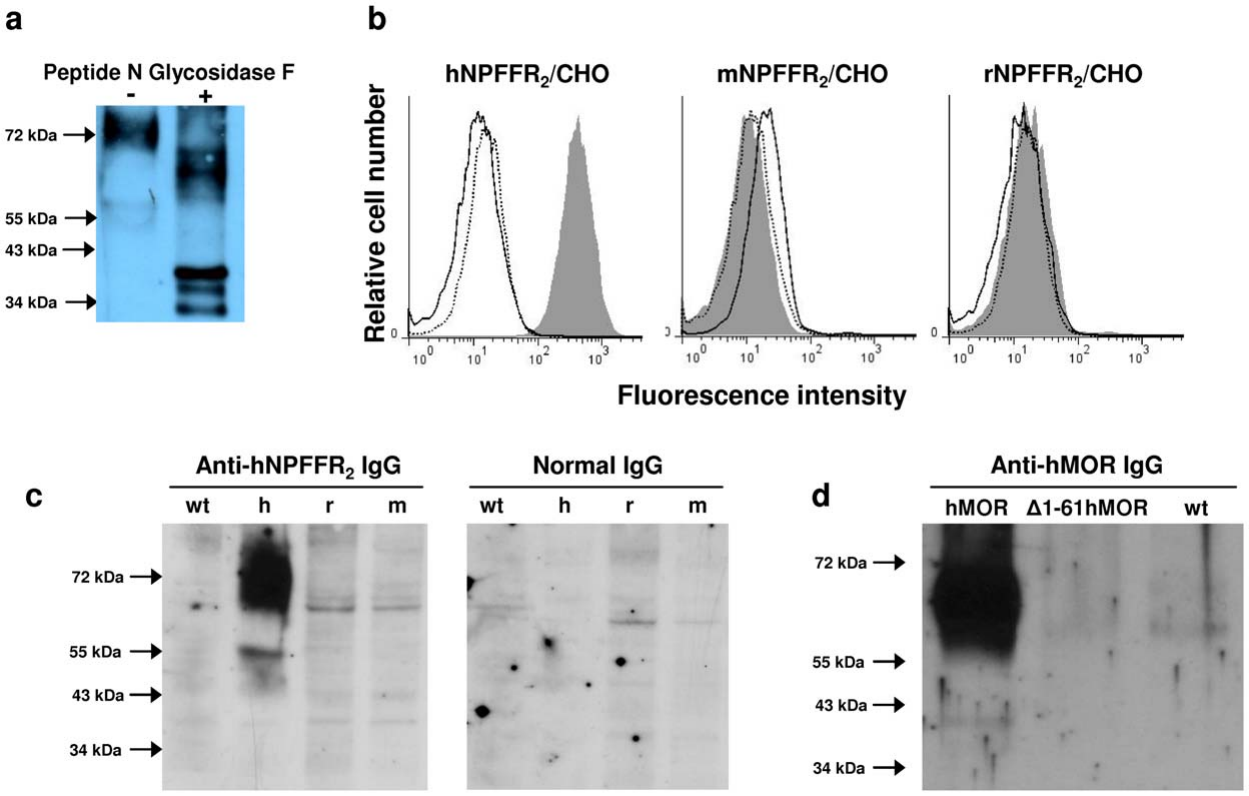
Specificity of anti-GPCR serum IgG. (**a**) Anti-hNPFFR_2_ IgG binding to deglycosylated hNPFFR_2_ receptor. Total cell membrane prepared from hNPFFR_2_-expressing CHO cells were incubated (+) or not (−) with Peptide N Glycosidase F enzyme for 1 hour at 37°C. Membrane protein extracts were run on SDS-polyacrylamide gels and then probed with anti-hNPFFR_2_ serum IgG after transfer onto PVDF membrane. The ability of anti-hNPFFR_2_ antibodies to discriminate human NPFFR_2_ used for immunization from highly homologous NPFFR_2_ of murine origin was examined by cytofluorometry (**b**) and Western-blotting (**c**). In (**b**), anti-hNPFFR_2_ serum IgG were incubated with wild-type CHO-K1 cells (open histogram) or CHO cells expressing NPFFR_2_ (grey histogram) originating from human (hNPFFR_2_/CHO, left panel), mouse (mNPFFR_2_/CHO, middle panel) and rat (rNPFFR_2_/CHO, right panel) for 30 min at 4°C. Bound IgG were then revealed with biotin-conjugated goat anti-mouse antibodies followed by an additional incubation with allophycocyanin-labeled streptavidin. Staining of NPFFR_2_-expressing CHO cells by control serum IgG from normal non-immunized mice is shown in dotted lines. The figure shows one representative experiment out of 3 performed. In (**c**), membrane protein lysates from wild-type CHO-K1 (wt), hNPFFR_2_/CHO (h), mNPFFR_2_/CHO (m) and rNPFFR_2_/CHO (r) were run on SDS-polyacrylamide gels and then probed with either anti-hNPFFR_2_ serum IgG (left panel) or normal serum IgG from non-immunized mouse (right panel) after transfer onto PVDF membrane. (**d**) The ability of anti-hMOR antibodies to bind to NH_2_-terminal extracellular segment of the hMOR was determined by Western-blotting. Cell membrane proteins from wild-type CHO-K1 (wt) and CHO cells expressing full-length (hMOR) or NH_2_-terminal truncated (Δ1-61hMOR) human MOR were probed with anti-hMOR serum IgG.

**Figure 4:**
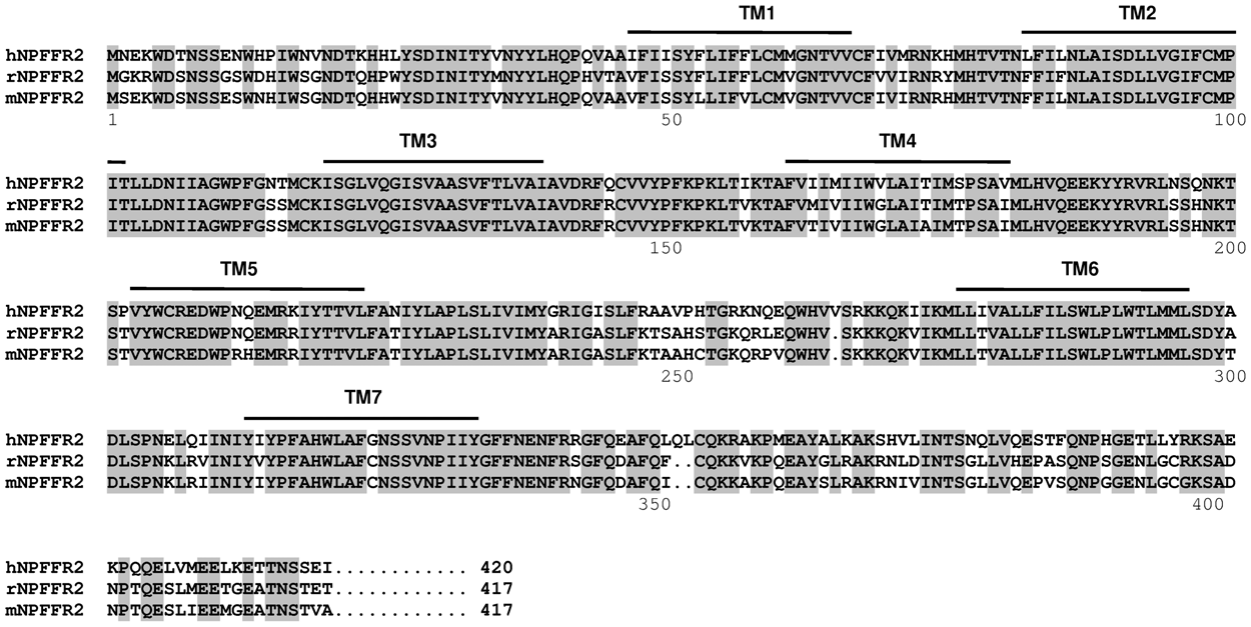
Amino acid sequence alignment of neuropeptide FF receptors 2 from Human, rat and mouse. Amino acid sequences of NPFF receptors 2 from Human (hNPFFR_2_), rat (rNPFFR_2_) and mouse (mNPFFR_2_) were compared for their amino acid sequence identities. Amino acid residues conserved (identical) across all the three species are enclosed in grey boxes. Putative transmembrane segments (TM) are indicated by bold lines above the sequence.

The apparent molecular weights of all the three receptors, revealed by immune sera as a unique band, were higher than theoretical ones or those observed when receptors originated from yeast. Bands were observed respectively at 80 kDa, 60 kDa and 70 kDa for hNPFFR_2_, hKOR and hMOR expressed on CHO cell membranes while their theoretical molecular weights calculated from the standard atomic weights are 49 kDa, 43 kDa and 45 kDa (**Table 1**). The discrepancy between the theoretical molecular weights of the receptors and the molecular weights corresponding to the bands revealed by anti-GPCR antibodies suggested that the receptors were probably glycosylated in CHO mammalian cells, as already described for many other GPCRs [32], [33]. This assumption was validated by deglycosylating the hNPFFR_2_ receptor with Peptide N Glycosidase F, which cleaves asparagine-linked oligosaccharides from glycoproteins, prior assessing it by western-blotting. As shown in figure 3a, anti-hNPFFR_2_ IgG antibodies revealed, in addition to the band at 80 kDa, others bands with lower apparent molecular weights. Thus, as exemplified for hNPFFR_2_, anti-GPCR polyclonal antibodies may recognize receptors with and without N-glycans. Anti-GPCR immune sera were also able to recognize receptors in their native conformation at the membrane surface of CHO cells as assessed by confocal microscopy (**Fig. 2b**) and cytofluorometry (**Fig. 2c**). Each immune serum IgG stained CHO cells expressing recombinant wild-type receptors but not control CHO-K1 cells (**Fig. 2**). Normal serum IgG from non-immunized mice, used as control, bound neither to wild type CHO-K1 cells nor to GPCR-expressing cells (**Fig. 2c**). The ability of anti-hNPFFR2 IgG to discriminate receptors with high amino acid sequence homology was evaluated by cytofluorometry. Although hNPFFR_2_ receptors originating from human, mouse and rat display more than 77% amino acid sequence identity [34] (**Fig. 4**), anti-hNPFFR_2_ antibodies only bound to human NPFFR_2_ that has been used to immunized animals (**Fig. 3b**). Similar results were obtained in western-blotting experiments (**Fig. 3c**). Thus, the anti-GPCR antibodies produced by using full-length GPCR molecules as immunogens, display a strong discriminative potency that allows them to distinguish between structurally similar molecules. Moreover, as exemplified with anti-hMOR antibodies that recognize the full-length but not the NH_2_ terminal-truncated form of the hMOR (**Fig. 3d**), antibodies might rather recognize extracellular domains of GPCRs.

### Detection of endogenous receptors

**Figure 5:**
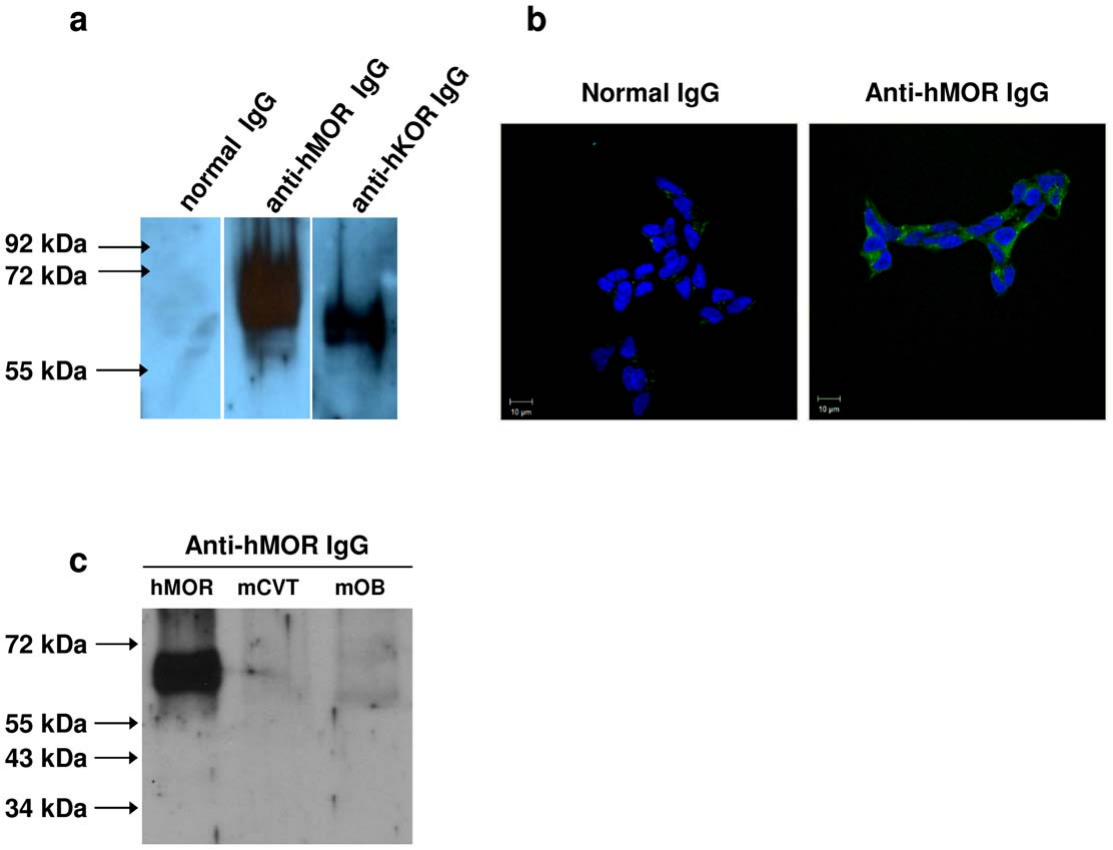
Binding of immune serum IgG to endogenously expressed GPCRs. In (**a**), protein lysates prepared from human spermatozoids were run on SDS-polyacrylamide gels and then probed with either control serum IgG from normal non-immunized mice, anti-hMOR serum IgG or anti-hKOR serum IgG after transfer onto PVDF membrane. In (**b**), human SH-SY5Y neuroblastoma cells were permeabilized with 0.05% Triton ×100 prior to be incubated with anti-hMOR serum IgG. Bound IgG were revealed with Alexa 488-labeled goat anti-mouse IgG antibodies (green staining). Cell nuclei were stained in blue with DAPI. Fluorescence images were acquired by confocal microscopy. Original magnification ×63. (**c**) The cross-reactivity of anti-hMOR antibodies towards murine MOR-containing tissue-extract antigens was examined by Western-blotting. Membrane protein lysates from hMOR/CHO (hMOR), mouse cerebellum (mCVT) and mouse olfactory bulb (mOB) were run on SDS-polyacrylamide gels and then probed with anti-hMOR serum IgG.

The ability of anti-GPCR IgG antibodies to recognize endogenous receptors was then investigated in human spermatozoids and human SH-SY5Y neuroblastoma cells in which the expression of MOR or KOR is well established [35], [36], [37]. As assessed by western-blotting on protein extracts from human spermatozoa, anti-hMOR as well as anti-hKOR serum IgG antibodies (dilution 1/2000) revealed only one band at the expected molecular weight (**Fig. 5a**). No band was revealed with control serum IgG from normal non-immunized mice used at the same dilution (**Fig. 5a**). Anti-hMOR serum IgG also revealed receptors endogenously expressed in SH-SY5Y neuroblastoma cells as assessed by immunocytofluorescence (**Fig. 5b**). Anti-hMOR antibody staining of SH-SY5Y neuroblastoma cells, revealed receptors expressed within the cytoplasm rather than at the membrane cell surface. This cellular distribution of MOR that contrasts with that observed in hMOR-expressing CHO cells was previously described in neurons [38]. The expression level of MOR in SH-SY5Y cells was then determined by using the MOR-selective opioid ligand [D-Ala2, N-Me-Phe4,Gly5-ol]-enkephalin (DAMGO). Binding assays performed on proteins extracted from crude membrane preparations including membranes from organelles, indicated that anti-hMOR IgG antibodies may detect receptors expressed at 0.04 pmol/mg of membrane proteins. As shown in figure 5c, anti-hMOR antibodies did not exhibit cross-reactivity towards mouse tissue extracts including MOR-expressing tissue such as olfactory bulb and cerebellum.

## Discussion

Our data indicate that immunization with functional native-like GPCRs is not required to generate specific antibodies able to recognize GPCRs in both native and denaturated forms. Anti-GPCR antibodies generated against SDS-solubilized or lyophilized proteins recognize native receptors expressed at the membrane surface of living cells (cytofluorometry) as well as denaturated/unfolded receptors (western-blotting and confocal microscopy). The antigen-binding site of antibodies may be conformational (*i.e.* dependent on receptor folding) or linear (*i.e.* dependent on primary sequence). Contrasting with linear epitopes that are exposed on unfolded receptor, conformational antigenic determinants accessible on native receptor are usually lost upon denaturation. However, as we have previously shown for MOR [39], [40], SDS-solubilized GPCRs display true helical secondary structures. SDS-solubilized GPCRs are probably not fully unfolded, but rather partially pre-folded, at least as far as the secondary structure is considered [26]. Alternatively, it could be hypothesized that n-alkanes (mineral oil) and D-dianhydromannitol monooleate (amphiphilic detergent) within Freund's adjuvant partially restore receptor structural architecture by mimicking molecular interactions of the lipid bilayer cell membrane with receptors. Thus, polyclonal anti-GPCR IgG antibodies may include both antibodies able to bind to linear epitopes accessible in both native and denatured forms of the receptor and antibodies able to bind to conformational antigenic determinants preserved in SDS-solubilized receptors or reconstituted in Freund's adjuvant (lyophilized receptor).

Taken together, our results show that the methodology developed to produce high amounts of purified GPCRs for structural studies is also valuable to generate highly specific anti-GPCR antibodies. This new strategy, that may be applicable in most laboratories, does not require receptors in native conformation to immunize animals nor an antibody purification step. Moreover, the method offers some other advantages including production of large quantities of receptors to immunize numerous animals or lyophilization suitable for a long storage period.

## Methods

### Ethics Statement

All ethics statements and consent were written and signed. Experiments that involved animal use were performed in compliance with the relevant laws and institutional guidelines (INSERM) and were approved by the local ethics committee (Midi-Pyrénées, France). All sperm donors were healthy and normozoospermic according to World Health Organization standards. Ethical approval was obtained from the Ethics Committee of the CECOS Midi-Pyrénées. Informed consent was obtained from all donors.

#### Preparation of recombinant G-protein coupled receptors

GPCR-cmyc-6his plasmid constructs (hMOR-cmyc-6his, hKOR-cmyc-6his, hNPFFR_2_-cmyc-6his), receptor overexpression in *Pichia pastoris* and preparation of GPCR-enriched fractions have already been described [30]. Briefly, after induction of GPCR expression with 0.5% methanol (v/v), GPCR-expressing yeast cells were harvested and broken with glass beads in 10 mM Tris–HCl, pH 7.5 containing protease inhibitors for 30 min at 4°C. Cell lysate was centrifuged at 1000 *g* for 15 min to remove unbroken cells and particulate matter. Supernatant was then centrifuged at 10,000 g for 30 min and the pellet containing the crude GPCR-containing fraction was stored at −20°C. Receptors were solubilized by diluting crude extracts in solubilization buffer (100 mM NaH_2_PO_4_, 10 mM Tris–HCl, 20 mM β-mercaptoethanol, 8 M urea, 0.1% SDS, pH 8) for 1 h at room temperature, with gentle agitation, on a rotating wheel. Solubilized receptors were incubated with Ni-chelated Sepharose Fast Flow (GE Healthcare Life Science, Uppsala, Sweden) for 1 h at room temperature. The sepharose resin was washed twice with the solubilization buffer first with and then without urea and β-mercaptoethanol. Ni-bound GPCR were eluted with a buffer containing 300 mM imidazole, 100 mM NaH_2_PO_4_, 10 mM Tris–HCl, 0.1% SDS, pH 8. Purity of the GPCR-enriched samples was assessed by silver nitrate staining and anti-c-myc Western-blotting. Then, GPCR preparations were either extensively dialysized against pure water and lyophilized or concentrated on a Centricon plus-20 centrifugal filter (Amicon, Millipore Corporation, MA).

#### Immunization of mice

Experiments were performed in compliance with the relevant laws and institutional guidelines (INSERM) and were approved by the local ethics committee (Midi-Pyrénées, France). Eight-week-old BALB/c mice (Janvier, Le Genest Saint Isle, France) were injected subcutaneously with 100 µg of purified GPCR (lyophilized or solubilised in 0.1% SDS) emulsified in complete Freund's adjuvant (Difco, Detroit, MI). Two subsequent injections two weeks apart were performed with same amounts of GPCR in incomplete Freund's adjuvant. Blood samples were collected by cardiac puncture under general anesthesia.

#### Cell culture and preparation of eukaryotic cell membrane

CHO-K1 cells expressing unmodified GPCRs including hMOR/CHO, hKOR/CHO, hNPFFR_2_/CHO, mNPFFR_2_/CHO, rNPFFR_2_/CHO or the hMOR deleted for the first 61 amino acids of the extracellular NH_2_-terminal segment (Δ1-61hMOR) [41], [42] were grown in high glucose DMEM (Invitrogen Corporation, Carlsbad, CA) supplemented with 10% fetal calf serum (FCS), 50 µg/ml gentamicine and 400 µg/ml geneticin G-418 sulfate to maintain GPCR-expressing cell selection. Wild-type CHO-K1 cells [43] and the human neuroblastoma SH-SY5Y cell line [44] were grown in the same medium without selective antibiotics. For the preparation of membranes, cells were harvested in phosphate buffer saline (PBS), frozen at −70°C for at least 1 h and then homogenized in 50 mM Tris–HCl, pH 7.5 using a Potter Elvehjem tissue grinder. The homogenate was centrifuged at 1000 *g* for 15 min at 4°C to discard residual cells, nuclei and mitochondria. The membrane fraction was then collected upon supernatant centrifugation at 100,000 *g* for 30 min at 4°C. The pellet was resuspended in Tris-HCl 50 mM, pH 7.4 and stored at −80°C after determination of the protein content.

#### Ligand-binding assay

Binding parameters were determined on membrane preparations by using tritiated MOR agonist, [^3^H]-DAMGO 50 Ci/mmol (1.85 TBq/mmol), (Perkin Elmer, Boston, MA,. Membranes (1–10 µg) were suspended in 50 mM Tris–HCl, 0.1% bovine serum albumin (BSA), pH 7.4 and binding was determined by adding increasing amounts of radiolabeled ligands. Non-specific binding was determined in the presence of unlabeled opioid antagonist, naloxone. After incubation for 1 h at 25°C, free ligands were removed by rapidly filtering the samples on Whatman GF/B filters, prior incubation in 0.3% polyethylenimine. The filters were rinsed three times with 4 ml of ice cold 10 mM Tris–HCl, pH 7.4 buffer containing 0.1% BSA, dried and bound radioactivity was counted in a Packard liquid scintillation spectrophotometric counter. Non-linear regression analyses of the data were performed using Prism 4.0 software (GraphPad Inc., USA).

#### Western blot analysis

Protein lysates prepared from *Pichia pastoris* cell extracts, GPCR-expressing CHO membranes, mouse olfactory bulb and cerebellum cell membranes or human spermatozoa were run on sodium dodecyl sulfate (SDS) 10% polyacrylamide gel electrophoresis and transferred onto a PVDF immun-blot membrane (Bio-Rad Laboratories, Hercules, CA). Receptors were probed with GPCR-primed mouse immune sera or when indicated with anti-c-myc monoclonal antibody (clone 9E10, diluted at 1∶1000, Sigma). Bound IgG were revealed using horseradish peroxidase-labeled sheep anti-mouse IgG antibodies (diluted at 1∶10000, Jackson Immunoresearch Lab., West Grove, PA). Signals were revealed with enhanced chemi-luminescence assay (ECL, Amersham Biosciences, GE Healthcare).

#### Cytofluorometric analysis

CHO-K1 or GPCR-expressing CHO cells were incubated with serum from either naïve non-immunized normal mice (background) or GPCR-primed mice for 30 min at 4°C in PBS containing 1% FCS and 2 mM EDTA. After washing, bound IgG were revealed with biotin-conjugated goat anti-mouse IgG antibodies (1/200; Jackson Immunoresearch Lab.) followed by an additional incubation with Allophycocyanin-labeled streptavidin (PharMingen, BD Biosciences, San Jose, CA). Data were collected on 10,000 living cells by forward and side scatter intensity on an FACs calibur flow cytometer (Becton Dickinson, Franklin Lakes, NJ), and were subsequently analyzed using the Flow Jo software (Tree Star Inc., Ashland, OR).

#### Immunofluorescence microscopy

CHO-K1 cells, GPCR-expressing CHO cells or human SH-SY5Y neuroblastoma cells were grown on sterile glass coverslips and fixed with 4% paraformaldehyde for 10 min at room temperature followed by 50 mM NH_4_Cl for 2 min. Cells were then incubated with normal or GPCR-primed mouse immune sera diluted in PBS (1/100 ), 2% FCS for 1 h at room temperature followed by an additional incubation with 1/500 Alexa Fluor 488-labelled goat anti-mouse IgG F(ab′)_2_-specific antibodies (Molecular Probes Inc., Eugene, OR) for 30 min at room temperature. Cells were permeabilized in PBS containing 0.05% Triton X-100 and nuclei were stained with DAPI (1/100) or with propidium iodide (1 µg/ml) in PBS containing 100 µg/ml of RNAseA for 10 min at room temperature. Coverslips were mounted using Vectashield medium (Vector Laboratories, CA). Fluorescence images were taken using an upright laser scanner confocal microscope (Leica TCS SP2, Germany) with ×100 oil immersion objective.

#### Sperm preparation

Human semen was obtained by masturbation after 2–3 days of abstinence. All sperm donors were healthy and normozoospermic according to World Health Organization standards. Ethical approval was obtained from the Ethics Committee of the CECOS Midi-Pyrénées. Informed consent was obtained from all donors. Samples were ejaculated into sterile containers and allowed to liquefy for 30 min at 37C° before processing.
